# Effect of Glutathione on Sperm Quality in Guanzhong Dairy Goat Sperm During Cryopreservation

**DOI:** 10.3389/fvets.2021.771440

**Published:** 2021-11-18

**Authors:** Jiahao Zou, Lixuan Wei, Dexian Li, Yongtao Zhang, Guang Wang, Lei Zhang, Ping Cao, Shaohua Yang, Guang Li

**Affiliations:** ^1^College of Animal Science and Technology, Northwest A&F University, Yangling, China; ^2^Xi'an Dairy Cow Breeding Center, Xi'an Agriculture and Rural Affairs Bureau, Xi'an, China; ^3^Longxian Animal Production Bureau, Baoji, China

**Keywords:** Guanzhong dairy goat, GSH, cryopreservation, sperm, quality

## Abstract

In the process of cryopreservation of dairy goat semen, it will face many threats such as oxidative damage, which will affect the motility and plasma membrane function of sperm. As an endogenous antioxidant in animals, glutathione (GSH) can significantly improve the quality of thawed sperm when added to the frozen diluent of semen of pigs and cattle. In this study, different concentration gradients of GSH [0 mmol/L (control), 1, 2, 3, 4 mmol/L] were added to the frozen diluent of Guanzhong dairy goat semen. By detecting the sperm motility parameters, acrosome intact rate and plasma membrane intact rate after thawing, the effect of GSH on the cryopreservation of dairy goat semen was explored. Sperm motility parameters were measured with the computer-aided sperm analysis (CASA) system (total power, TM; forward power, PM; linearity, LIN; average path speed, VAP; straight line speed, VSL; curve speed, VCL; beat cross frequency, BCF). The sperm acrosome integrity rate after thawing was detected by a specific fluorescent probe (isothiocyanate-labeled peanut agglutinin, FITC-PNA), and the sperm plasma membrane integrity rate after thawing was detected by the hypotonic sperm swelling (HOST) method. Reactive oxygen species (ROS) kit, malondialdehyde (MDA) kit, superoxide dismutase (SOD) kit, glutathione peroxidase (GSH-PX) kit were used to detect various antioxidant indicators of thawed sperm. *in vitro* fertilization experiment was used to verify the effect of adding glutathione on sperm fertilization and embryo development. The results showed that when the concentration of glutathione was 2 mmol/l, the sperm viability, plasma membrane intact rate, and acrosome intact rate were the highest after thawing, reaching 62.14, 37.62, and 70.87% respectively, and they were all significantly higher. In terms of antioxidant indexes; the values of SOD and GSH-PX were 212.60 U/ml and 125.04 U/L, respectively, which were significantly higher than those of the control group; The values of ROS and MDA were 363.05 U/ml and 7.02 nmol/L, respectively, which were significantly lower than the control group. The addition of 2 mmol/L glutathione significantly improves the fertilization ability of sperm. In short, adding 2 mmol/l glutathione to the semen diluent can improve the quality of frozen Guanzhong dairy goat sperm.

## Introduction

With the development of modern animal husbandry, artificial insemination technology has been widely used. The cryopreservation of dairy goat semen is beneficial to the long-term storage of the fine-bred dairy goat semen and long-distance transportation in different places, which helps to promote the process of improving the dairy goat industry and improve economic benefits (1). However, the research on cryopreservation of Guanzhong dairy goat semen has problems such as low sperm viability and low conception rate after thawing. Frozen semen has not been widely used in production. Studies have found that during the preservation of semen, sperm will produce reactive oxygen species (ROS) due to respiratory metabolism (2). High concentrations of ROS will destroy the plasma membrane structure of sperm and cause oxidative damage to sperm, thereby reducing sperm fertilization (3). Therefore, it is very necessary to add antioxidants to the frozen diluent of Guanzhong dairy goat sperm.

Glutathione (GSH) is a tripeptide compound formed by the condensation of glutamic acid, cysteine, and glycine in the form of peptide bonds. Glutathione is an important endogenous antioxidant in animals. It is one of the ROS scavengers existing in sperm and seminal plasma. It plays an important role in the defense mechanism of protecting sperm against oxidative damage (4, 5). At present, studies have shown that adding a certain amount of GSH to the cryopreservation of semen of pig, cattle, sheep, and Chios ram can significantly improve the fertilization ability of sperm after thawing (2, 6–9). It is not clear whether the use of GSH in the cryopreservation of Guanzhong dairy goat semen will affect the quality of sperm after thawing. Therefore, the purpose of this study was to determine the effect of adding GSH on sperm quality in frozen Guanzhong dairy goat semen.

## Materials and Methods

In this study, all goats used in the experiment were raised in accordance with the No. 5 Announcement of the Ministry of Agriculture, P. R. China. All procedures in our animal research have been approved by the Animal Protection and Use Committee of Northwest A&F University (Yangling, China) (license number: 17-347, data: October 13, 2017).

### Animals

The semen used in the experiment was collected from six healthy Guanzhong dairy goats for breeding in a certain goat farm in Lantian, Shaanxi Province. They were all 2.5–3 years old, in good health and strong sexual desire. This experiment was conducted between July 2020 and December 2020 (10).

### Semen Collection

The artificial vagina method was used to collect semen from dairy goats. The semen was sent to the laboratory immediately after collection, and the sperm sample was evaluated for sperm density and sperm motility at 35–38°C. Each ejaculate was processed and then analyzed individually. Sperm samples with normal morphology, deformity rate <5%, sperm motility >0.8, and density >1.5 billion/ml were used for subsequent experiments.

### Semen Processing

The semen that passed the microscopic examination was divided into four groups, and they were diluted isothermally with the first diluent with different concentrations of GSH, and placed in a refrigerator at 4°C for 2 h. The first diluent was composed of 30 g/L glucose, 48 g/L lactose, 16 g/L sodium citrate, 150 ml/L egg yolk, and 1 million IU/L penicillin and streptomycin. After the first equilibration was completed, perform the second isothermal dilution in the 4°C operating table, and then continued to equilibrate in the 4°C refrigerator for 2 h. The second diluent was composed of 30 g/L glucose, 48 g/L lactose, 16 g/L sodium citrate, 150 ml/L egg yolk, 6% glycerin, and 1 million IU/L penicillin and streptomycin. After the equilibration was completed, the semen was loaded into 0.25 ml thin tubes and the temperature was reduced from 5 to −140°C according to the specific freezing procedure. The specific freezing procedure is: cooling from 5 to −10°C at a rate of 6°C/min, and from −10 to −140°C at a rate of 40°C/min. Then the thin tube was immersed in liquid nitrogen at −196°C to complete the freezing process (11).

After thawing, the sperm motility, plasma membrane intact rate, acrosome intact rate, and antioxidant index were tested. In order to evaluate sperm motility, plasma membrane intact rate, acrosome intact, and antioxidant indexes, the samples were thawed in 38°C water bath for 35 s, and samples were taken for testing.

### Sperm Evaluation

#### Sperm Motility

The semen samples were evaluated by the computer-aided sperm analysis (CASA) system for sperm motility parameters. The following parameters were tested: total motility (TM, %), progressive motility (PM, %), linearity (LIN, %), average path velocity (VAP, μm/s), straight line velocity (VSL, μm/s), curvilinear velocity (VCL, μm/s), beat cross frequency (BCF, Hz).

#### Sperm Plasma Membrane and Acrosome Integrity

The integrity of the plasma membrane was tested by the hypotonic sperm swelling (HOST) method. The specific method is to take the thawed semen and mix it with a solution of hypotonic fructose and sodium citrate (the concentration of fructose used was 13.5 g/L and the concentration of citrate was 7.35 g/L), and incubate it at 37°C for 60 min. After incubation, observed the sample with a phase-contrast microscope (BX-60, Olympus, Tokyo, Japan) at ×1,000 times. If the sperm plasma membrane was intact, the head of the sperm will absorb water and swell, and the tail will swell and curl (1).

The acrosome integrity was detected by a specific fluorescent probe (isothiocyanate-labeled peanut agglutinin, FITC-PNA). The specific method is to completely mix the thawed sperm sample with ethD-1 in the kit (Purchased from Biyuntian Biological Company) at 37°C for 15 min. Put 5 μl of the mixture on a glass slide, drop it in 95% ethanol for 30 s, then add 15 μl of FITC-PNA solution, react at 4°C for 30 min, and then remove it by PBS (12). A fluorescence microscope (CX-31, Olympus, Tokyo, Japan) was used to evaluate the acrosome integrity at ×1,000 times. Two hundred spermatozoa were evaluated in five different areas.

#### Antioxidant Indexes

Reactive oxygen species kit (Purchased from Biyuntian Biological Company), malondialdehyde (MDA) kit (Purchased from Soleibao Biological Company), superoxide dismutase (SOD) kit (Purchased from Soleibao Biological Company), glutathione peroxidase (GSH-PX) kit (Purchased from Biyuntian Biological Company) were used to detect various antioxidant indicators of thawed sperm. Referred to the kit instructions for specific operation methods.

### *In vitro* Fertilization and Embryo Development

#### Collection and Culture of Oocytes

Goat ovaries without visible corpus luteum were harvested at the slaughterhouse and transported to the laboratory in 0.9% sodium chloride with 1 million IU/L penicillin and streptomycin. The collected ovaries were washed three times in sterile 0.9% sodium chloride and twice in phosphate buffered saline supplemented with 10% fetal bovine serum. Oocytes were aspirated from medium-sized follicles (3–6 mm in diameter) using a disposable syringe. The cumulus-oocyte complexes (COCs) with at least three complete and dense layers of cumulus cells and uniform granular cytoplasm were selected for *in vitro* maturation under a stereo microscope. Then the COCs were washed three times in DPBS, twice in the maturation medium (the medium was TCM-199 supplemented with 10% FBS, both FBS and TCM-199 were purchased from Thermo Fisher Scientific), and cultured in an incubator with a CO_2_ concentration of 5% at 38.5°C for 27 h. Cumulus-oocyte complexes with expanded cumulus clouds were selected for subsequent *in vitro* fertilization (13).

#### Sperm Processing Before *in vitro* Fertilization

About 100 μl of thawed semen was diluted with 5 ml sperm BO (Purchased from Amyjet Scientific) medium, and centrifuged (1,200 rpm) at 25°C for 5 min (14). The supernatant was discarded and the sperm pellet resuspended in the same medium. This step was repeated three times to remove all seminal plasma and frozen diluent. The obtained sperm precipitate was resuspended in BO medium and diluted 1:1 in BO medium containing 50 μg/ml heparin. Sperm was incubated in an incubator at 38.5°C and 5% CO_2_ for 1–2 h until they were used for *in vitro* fertilization.

#### *In vitro* Fertilization

After 27 h of *in vitro* maturation, the oocytes were transferred to 100 μl of BO medium (under paraffin oil) and the capacitated sperm suspension was added to the droplets to reach a final sperm concentration of 5 × 10^6^ motile cells/ml. *In vitro* fertilization was carried out in an incubator with 38.5°C and 5% CO_2_ concentration for 24 h.

#### *In vitro* Embryo Culture

Twenty-four hours after fertilization, the putative zygotes were washed in order to separate the adherent sperm cells. putative zygotes were placed in a CO_2_ incubator in embryonic development medium (MCR_2_aa medium containing 10% fetal bovine serum and 3 mg/ml bovine serum albumin) at 38.5°C and 5% CO_2_ concentration for 9 days.

#### Evaluation of Embryo Development *in vitro*

After insemination, half of the medium was supplemented with fresh medium every 48 h. The embryos were morphologically evaluated under an inverted phase-contrast microscope (BX-60, Olympus, Tokyo, Japan). The cleavage rate was measured 48 h after insemination, and the blastocyst rate was measured on the 9th day of embryo culture.

### Statistical Analysis

All data are expressed as mean ± standard deviation. SPSS26.0 was used for statistical analysis. Analysis of variance (ANOVA) and Duncan's multiple range test were used to locate differences, and the sperm quality and motility parameters, plasma membrane integrity, and acrosome integrity were compared between groups. The independent sample *t*-test was used to compare the cleavage rate and the blastocyst rate. Differences were considered significant at *P* < 0.05.

## Results

### Effect of Different Concentrations of Glutathione on Sperm Motility

It can be seen from Table 1 that after thawing, the parameters related to sperm motility performance in semen supplemented with 2 mmol/L glutathione were significantly higher than those of other groups (*P* < 0.05). In many quality parameters, there is no significant difference between the two groups with 1 and 3 mmol/L glutathione. But most of their parameters are significantly higher than the control group (*P* < 0.05). Most parameters related to sperm motility in semen with 4 mmol/L glutathione were significantly lower than other groups (*P* < 0.05).

**Table 1:** Effect of glutathione (GSH) on sperm motility parameters of Guanzhong dairy goats after thawing.

**GSH (mmol/L)**	**Sperm motility parameters**						
	**TM (%)**	**PM (%)**	**VAP (μm/s)**	**VSL (μm/s)**	**VCL (μm/s)**	**LIN (%)**	**BCF (Hz)**
0	57.15 ± 0.53^a^	21.56 ± 0.52^a^	35.12 ± 0.47^a^	24.67 ± 0.41^a^	58.70 ± 0.52^a^	42.04 ± 0.71^a^	27.43 ± 0.29^a^
1	59.23 ± 0.75^b^	23.40 ± 0.31^b^	36.12 ± 0.32^b^	26.71 ± 0.54^b^	61.16 ± 0.32^b^	43.67 ± 1.04^b^	28.57 ± 0.39^b^
2	62.14 ± 0.58^c^	25.47 ± 0.64^c^	37.66 ± 0.46^c^	31.42 ± 0.61^c^	63.97 ± 0.41^c^	49.12 ± 0.97^c^	31.01 ± 0.65^c^
3	58.75 ± 0.51^b^	23.11 ± 0.66^b^	36.08 ± 0.35^b^	28.18 ± 0.40^d^	61.51 ± 0.37^b^	45.81 ± 0.66^d^	29.20 ± 0.34^b^
4	56.00 ± 0.60^d^	19.89 ± 0.51^d^	34.26 ± 0.30^d^	25.54 ± 0.64^e^	58.04 ± 0.29^d^	44.00 ± 1.12^b^	27.40 ± 0.58^a^

*TM, total motility; PM, progressive motility; VAP, average path velocity; VSL, straight line velocity; VCL, curvilinear velocity; LIN, linearity; BCF, beat cross frequency. Three repeats were set for each group*.

a−e *Within a column, means without a common superscript differed (P < 0.05)*.

### Effect of Different Concentrations of Glutathione on Sperm Plasma Membrane and Acrosome Integrity

Analysis of the data in Table 2 shows that after thawing, the acrosome integrity and plasma membrane integrity of sperm in the semen with 2 mmol/L glutathione were significantly higher than other groups (*P* < 0.05). There was no significant difference between the two groups that added glutathione at 1 and 3 mmol/L, but they were all significantly higher than the control group (*P* < 0.05). The acrosome integrity and plasma membrane integrity of sperm in semen supplemented with 4 mmol/L glutathione were not significantly different from those in the control group.

**Table 2:** Effect of glutathione (GSH) on the sperm acrosome and plasma membrane integrity of Guanzhong dairy goats after thawing.

**GSH (mmol/L)**	**0**	**1**	**2**	**3**	**4**
**Acrosome integrity (%)**	67.51 ± 0.52^a^	69.78 ± 0.27^b^	70.87 ± 0.24^c^	69.83 ± 0.48^b^	67.69 ± 0.33^a^
**Plasma membrane integrity (%)**	35.77 ± 0.28^a^	36.58 ± 0.28^b^	37.62 ± 0.30^c^	36.60 ± 0.55^b^	35.55 ± 0.36^a^

*Three repeats were set for each group*.

a−c *Within a line, means without a common superscript differed (P < 0.05)*.

### Effect of Different Concentrations of Glutathione on Sperm Antioxidant Indicators

As shown in Table 3, after thawing, when the concentration of glutathione was 2 mmol/l, the activities of SOD and GSH-PX in semen were significantly higher than those of other groups (*P* < 0.05), and the values of ROS and MDA were significantly lower than those of other groups (*P* < 0.05). When the glutathione concentration reached 4 mmol/l, the activities of SOD and GSH-PX in semen after thawing were significantly lower than those of the control group (*P* < 0.05), and the values of ROS and MDA were significantly higher than those of the control group (*P* < 0.05).

**Table 3:** Effect of glutathione (GSH) on sperm antioxidant indicators of Guanzhong dairy goats after thawing.

**GSH (mmol/L)**	**Sperm antioxidant indicators**			
	**SOD (U/ml)**	**GSH-PX (U/L)**	**ROS (U/ml)**	**MDA (nmol/L)**
0	175.99 ± 3.82^a^	106.30 ± 2.95^a^	482.28 ± 7.20^a^	6.28 ± 0.14^a^
1	198.07 ± 2.99^b^	118.77 ± 2.73^b^	440.11 ± 7.39^b^	5.55 ± 0.13^b^
2	218.23 ± 4.04^c^	133.63 ± 3.12^c^	395.58 ± 7.42^c^	5.05 ± 0.17^c^
3	181.42 ± 4.52^d^	108.97 ± 4.12^a^	465.03 ± 7.93^d^	5.99 ± 0.14^d^
4	154.47 ± 3.55^e^	96.59 ± 3.20^d^	505.71 ± 5.68^e^	6.47 ± 0.11^e^

*Three repeats were set for each group*.

a−e *Within a column, means without a common superscript differed (P < 0.05)*.

### Effect of Glutathione (2 mmol/L) on *in vitro* Fertilization and Embryo Development of Dairy Goat Oocytes

Because in the previous study 2 mmol/L GSH concentration better improved sperm parameters, in the *in vitro* fertilization and embryo development studies only the effects of the two groups with 2 mmol/L GSH and without GSH (control) were compared. It can be seen from Table 4 that the cleavage rate measured 48 h after *In vitro* fertilization, the group with 2 mmol/L glutathione was significantly higher than the group without glutathione (*P* < 0.05). The blastocyst rate measured on the 9th day of embryo culture, the group with 2 mmol/L glutathione was also significantly higher than the group without glutathione (*P* < 0.05).

**Table 4:** Effect of glutathione (2 mmol/L) on *in vitro* fertilization and embryo development of dairy goat oocytes.

**GSH (mmol/L)**	**Total number**	**Cleavage**	**Blastocyst**
	**of oocytes**	**rate (%)**	**rate (%)**
0	457	56.65 ± 1.53^a^	22.93 ± 1.15^a^
2	401	69.57 ± 0.81^b^	37.15 ± 0.81^b^

*Three repeats were set for each group*.

a,b *Within a column, means without a common superscript differed (P < 0.05)*.

## Discussion

In the process of cryopreservation of sperm, there are many threats such as temperature changes, osmotic pressure changes, ice crystal damage, and oxidative damage. Under normal conditions, sperm oxidation and anti-oxidation are in a dynamic balance, but sperm will die and be damaged during freezing. More ROS and reduced antioxidant enzyme activity in ultra-low temperature environment, which leads to increased threat of oxidative damage to sperm. Therefore, supplementation of exogenous antioxidant substances in cryo-diluent is very important for the cryopreservation of semen (15, 16).

In this study, glutathione was used in the diluent of Guanzhong dairy goat semen for the first time, and it clarified the importance of adding glutathione during the cryopreservation of semen to improve the quality of dairy goat semen. From the results of the experiment, adding 2 mmol/l glutathione to the frozen dilution of dairy goats can significantly improve the motility of sperm and plasma membrane and acrosome integrity. At the same time, it can significantly increase the antioxidant level of sperm.

Sperm motility is closely related to reproductive efficiency. The motility of sperm after thawing is one of the important predictors of cryopreservation and fertilization. The results of this study showed that when the concentration of glutathione in the diluted solution was 2 mmol/L, the motility performance of the sperm after thawing was significantly improved, and the higher concentration of glutathione had a negative effect on the motility of the sperm after thawing. This is the same as the results of studies on cattle and pigs (17–19).

The integrity of the plasma membrane and acrosome of sperm directly affect the fertilization ability of sperm. The results of this study showed that when the concentration of glutathione added in the diluent was 2 mmol/L, the integrity of the plasma membrane and acrosome of the sperm after thawing was significantly improved. Similarly, studies in mice and dogs have also confirmed that glutathione can improve sperm plasma membrane function (4, 20). In addition, high concentrations of antioxidants have no significant effect on the plasma membrane function of dairy goat sperm after thawing. This may be because the addition of high concentrations of glutathione increases the osmotic pressure of the diluent, which adversely affects the stability of sperm structure (21).

A series of operations such as cooling, freezing, and thawing can cause physical, chemical, and oxidative damage to sperm. Among them, oxidative damage mainly comes from ROS (22). Studies have shown that low concentrations of ROS have a positive effect on the normal development of sperm and the process of fertilization, but if the total amount of ROS exceeds the decomposing power of the antioxidant enzymes in the sperm, lipid peroxidation will occur (23–25). Lipid peroxidation can cause irreversible oxidative damage to sperm and affect the pregnancy rate. Malondialdehyde is a substance produced by lipid peroxidation. It has toxic side effects on sperm, which will lead to the decline of sperm quality. The semen of healthy male animals has an antioxidant enzyme system that resists ROS. Glutathione peroxidase and SOD can make the production and clearance of ROS in a dynamic balance, and effectively protect the semen from ROS attack. It has been proved that during the freezing process of semen, supplementation of exogenous antioxidants can enhance the ability of sperm to resist peroxidation and reduce the damage caused by it (26). Glutathione, as an endogenous antioxidant in animals, has strong antioxidant and free radical scavenging capabilities. In this experiment, after adding 2 mmol/L of glutathione to the diluent, the SOD and GSH-PX content in the sperm were increased, while the contents of MDA and ROS decreased, indicating that the appropriate addition of glutathione is helpful to improve the antioxidant capacity of sperm. This is consistent with the effect of glutathione cryopreservation of pig semen (27).

The *in vitro* fertilization experiment can intuitively reflect the fertilization ability of sperm. The cleavage and blastocyst stages are important periods of embryonic development (28). By comparing the cleavage and blastocyst rates with 2 mmol/L glutathione and without glutathione, it can be seen from the comparison that the addition of 2 mmol/L glutathione improves the sperm fertilization ability.

## Conclusions

In summary, adding 2 mmol/L glutathione to the diluent of Guanzhong dairy goat semen has a good effect on frozen semen, significantly improving sperm motility and plasma membrane function, thereby improving the quality of semen after thawing.

## Data Availability Statement

The original contributions presented in the study are included in the article/supplementary material, further inquiries can be directed to the corresponding author/s.

## Ethics Statement

The animal study was reviewed and approved by the Animal Protection and Use Committee of Northwest A&F University. Written informed consent was obtained from the owners for the participation of their animals in this study.

## Author Contributions

GL put forward the design. LZ and PC revamped and improved experiment plan. JZ participated in the experiments and wrote the manuscript. Meanwhile, LW also participated in this experiment partly and analysis of data. DL, YZ, GW, and SY were involved in revising the manuscript. Finally, all authors read and agreed with the eventual manuscript.

## Funding

This research was supported by Major Science and Technology Project of Shaanxi Agricultural Collaborative Innovation and Extension Alliance (LMZD202002), Shaanxi Provincial Science and Technology Co-ordination Innovation Project (2018ZDCXL-NY-01-04), Shaanxi Agricultural Science and Technology Innovation and Extension Project (NYKJ-2019-YL16), and Xi'an Science and Technology Plan Project (20NYYF0033).

## Conflict of Interest

The authors declare that the research was conducted in the absence of any commercial or financial relationships that could be construed as a potential conflict of interest.

## Publisher's Note

All claims expressed in this article are solely those of the authors and do not necessarily represent those of their affiliated organizations, or those of the publisher, the editors and the reviewers. Any product that may be evaluated in this article, or claim that may be made by its manufacturer, is not guaranteed or endorsed by the publisher.
